# Homogeneous redox catalysed reduction of CO_2_ by nickel cyclam catalyst and chromium-based redox mediator

**DOI:** 10.1039/d5dt02738a

**Published:** 2026-04-24

**Authors:** Mark Potter, Kathryn E. Toghill

**Affiliations:** a Department of Chemistry, Lancaster University Lancaster LA1 4YB UK k.toghill@lancaster.ac.uk

## Abstract

The electrochemically decoupled reduction of CO_2_ using nickel cyclam as a homogeneous catalyst and chromium propanediaminetetraacetate (PDTA) as a reductive redox mediator is reported in aqueous media. Uniquely, the Ni cyclam catalyst, known for exclusive homogenous CO production instead gives a mixed product selection of HCOO(H) and CO depending on the supporting electrolyte composition, with KHCO_3_ favouring CO and KCl favouring HCOO(H). The CrPDTA shows no conventional electrochemical booster effects, but exclusively reacts with the Ni cyclam when generated as a bulk Cr(ii) species.

## Abstract 2

The electrochemically decoupled reduction of CO_2_ using nickel cyclam as a homogeneous catalyst and chromium propanediamine tetraacetate (PDTA) as a reductive redox mediator is reported in aqueous media.

## Graphical abstract

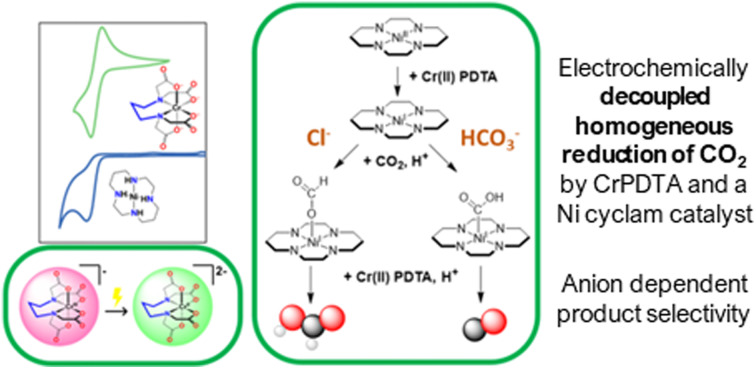

## Introduction

The use of redox mediators (RM) alongside homogeneous catalysts is a technique that is increasingly reported for a range of applications including: biosensing,^1–3^ water electrolysis,^4,5^ fuel cells,^6^ and batteries.^7,8^ More recently, the approach has been applied to electrochemical CO_2_ reduction (ECO_2_R).^9–11^ Akin to the enzyme-cofactor interactions observed in many biological and photosynthetic processes, a mediator can be used to transfer electrons (and possibly protons) to a catalytic site. Unsurprisingly, this was first reported for the ubiquitous iron porphyrin catalyst, known for excellent CO selectivity,^12,13^ utilising an NADH inspired RM to boost turnover frequency 13 times without effecting product selectivity.^9^ In this instance, the RM acted as a two-electron, two-proton source, enabling a concerted mechanism to product formation during controlled potential electrolysis. A second reported example used a dibenzothiophene-5,5-dioxide RM alongside a chromium based catalyst with a hydroxypyridine ligand in a process denoted co-catalysis.^10,11^ The RM delivered a single electron equivalent to the activated catalyst through an inner sphere mechanism relying on through-space electronic conjugation. In both of these studies the mediator facilitated the typical homogeneous electrocatalytic process with electrolysis potentials below −2 V *vs.* Fc^+^/Fc, where the redox mediator is acting as a true co-catalyst, actively participating in the product forming mechanism.

In this work, we report an aqueous chromium complex, Cr PDTA, as an RM in a *decoupled* electrochemical reduction reaction to drive directly the reduction of CO_2_ without the use of an electrode through a homogeneous reaction with a Ni cyclam catalyst. The objective was to use the Cr(ii) species as an electron source, producing a fully homogeneous reaction rather than a heterogeneous mediated reaction at an electrode. Here, the Cr(iii)PDTA is reduced to a Cr(ii) state in an electrochemical flow cell prior to the reaction with the Ni cyclam and CO_2_. Thereafter a separate reaction occurs between the charged RM, catalyst and substrate to yield reduction products and the discharged RM. We have previously reported this process for CO_2_ reduction on heterogeneous bismuth catalysts to formate,^14^ but here we successfully demonstrated its use alongside the Ni cyclam catalyst as an RM for homogeneous CO_2_ reduction in aqueous solutions.

## Results and discussion

**Fig. 1:**
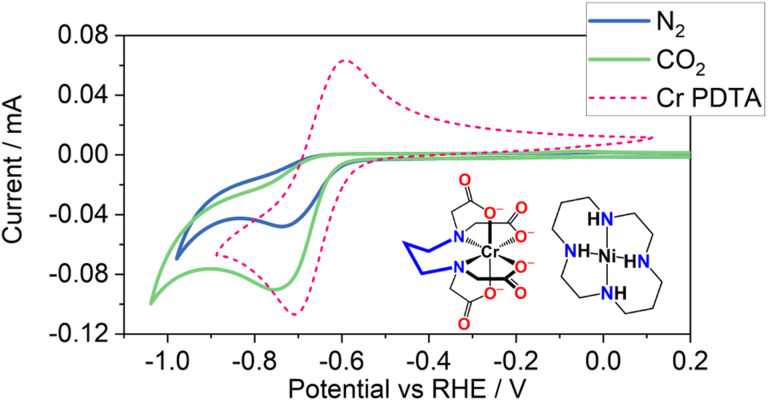
Cyclic voltammetry of 1 mM Ni(cyclam)Cl_2_ in 1 M KHCO_3_ under N_2_ (blue) and CO_2_ (green) atmospheres, using a glassy carbon working electrode at a scan rate of 100 mV s^−1^. Potential has been refenced against RHE using pH value of 8.5 for the N_2_ saturated electrolyte and 7.5 for the CO_2_ saturated electrolyte. Cyclic voltammetry of 10 mM Cr PDTA under N_2_ (pH 8.5) (dashed pink) is overlayed to show the overlap in potential of the two species. The structures of Ni cyclam and Cr PDTA are inset.

The electrochemical characteristics of nickel cyclam in non-aqueous solutions are well reported. Under inert conditions, two reversible redox couples are observed, Ni(iii)/Ni(ii) at 0.81 V *vs.* SHE and Ni(ii)/Ni(i) at −1.23 V *vs.* SHE.^15^ Ni cyclam has been reported for CO_2_ reduction in a number of solvents including DMF,^16^ MeCN,^15^ and water.^17^Fig. 1 shows the cyclic voltammetry of Ni cyclam in 1 M KHCO_3_ with and without CO_2_ (the equivalent in 1 M KCl is in Fig. S1). The Ni(ii)/Ni(i) couple is usually not observed as being reversible under aqueous conditions, due in part to onset of the hydrogen evolution rection (HER), which is more pronounced in the 1 M KCl solution (Fig. S1).^15^ Upon saturation with CO_2_ however, a significant increase in current is observed, which is indicative of a catalytic process, notably suppressing HER in the KCl solution, despite a lower pH caused by CO_2_ saturation. This wave at −0.6 V *vs.* RHE occurs at a similar potential to the Cr(iii)/Cr(ii) couple in the Cr PDTA mediator we have previously reported for decoupled electrochemical CO_2_ reduction (DECO_2_R), as shown in Fig. 1.^14^ This excellent overlap of potentials suggests that the two complexes could be paired together for homogeneous catalytic reduction. The potential overlap between the two species allows for efficient charge transfer between the reduced RM and the catalyst. RMs are commonly reported to lower the overpotential of a reaction to improve kinetics, however when used as a reducing equivalent in this way, the redox potential of the RM should ideally be more negative than that of the catalyst such that charge transfer occurs quickly.^18^ An excessively negative RM would result in lower energy efficiency, while an insufficiently negative RM would limit charge transfer onto the catalyst.

Cyclic voltammetry of an electrolyte containing both Cr PDTA and Ni cyclam (Fig. S3–S6) gave no clear indication of a co-catalytic process due to the already poor reversibility of the Cr PDTA voltammetry on glassy carbon. However, such an experiment was not anticipated to have a strong correlation, as while both the Ni cyclam and Cr PDTA are electroactive, only the Ni cyclam is CO_2_ active. The voltammetry was therefore dominated by the typical electrochemical redox behaviour of the Cr PDTA in greater excess. It should be noted that the small oxidation peak observed between the two primary redox events only occurs if Ni(i)/N(ii) redox has been performed and may correspond to the re-oxidation of a relatively stable Ni(i) complex or intermediate.

### Decoupled homogeneous electrolysis

Ni cyclam was first tested as a catalyst for DECO_2_R using Cr PDTA as an electron donating RM in a batch configuration. A 10 mL aliquot of 20 mM charged Cr(ii) PDTA (200 µmol) was added to a Schlenk flask containing 10 mL of 2 mM Ni(ii)cyclam (20 µmol) for a total of 20 mL of solution. This provides enough electrons for five turnovers of the catalyst assuming only two-electron products. faradaic yields were determined from the ratio of the total number of electrons needed to produce the measured products with the amount of charge theoretically provided by the Cr PDTA in solution being oxidised from Cr(ii) to Cr(iii), 200 μmol for 20 mL of 10 mM mediator.

**Fig. 2:**
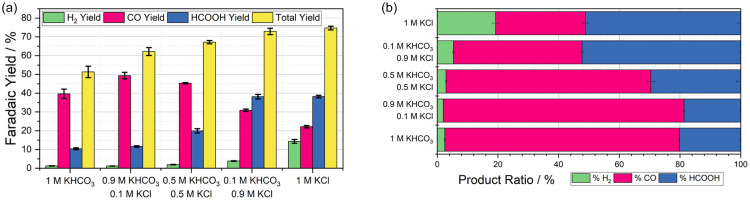
(a) Chart indicating the faradaic yield towards H_2_ (green), CO (pink), HCOOH (blue), and in total (yellow) for DECO_2_R using 1 mM Ni cyclam as a homogeneous catalyst and 10 mM Cr PDTA as an RM with various combinations of KHCO_3_ and KCl supporting electrolyte. (b) Chart indicating the relative selectivity towards the products towards H_2_ (green), CO (pink), HCOOH (blue) for this data.

The composition of the supporting electrolyte was varied, maintaining 1 M [K^+^] but changing the anion between Cl^−^ and HCO_3_^−^. This had considerable effect on both the overall faradaic yield (FY) of the reaction and the individual product selectivity, as shown in Fig. 2a. In all instances, three products of reductions were observed; CO, HCOO(H) and H_2_. This was unexpected, as Ni cyclam is typically a CO forming catalysts, with very few accounts of it producing formate.^19^ Overall, the FY of the decoupled reduction reaction varied from 52–77%. Replacing a portion of the HCO_3_^−^ with Cl^−^ resulted in a significant improvement in FY, with lower FY observed for pure carbonate buffer and highest FY observed for pure KCl.

Fig. 2b shows the ratio of products in each condition, and indicates that Ni cyclam was most selective towards CO production (77%) when 1 M KHCO_3_ was used as the supporting electrolyte, however a significant portion of HCOO(H) was still produced, more than expected based on the product selectivity reported in the existing literature.^19^ As the ratio of HCO_3_^−^ to Cl^−^ decreased the reaction became less selective, producing more HCOO(H). In the presence of HCO_3_^−^ HER constituted only a small portion of the FY (<5%). However, fully replacing the HCO_3_^−^ with Cl^−^ saw HCOO(H) become the major product and HER now contribute nearly 20% of the product selectivity and 15% of the FY. Control reactions confirmed that no CO_2_R products were formed when there was no source of CO_2_, instead a moderate amount of HER occurred, very slowly compared to the rate of discharge during CO_2_R, with a rate around 10× slower for a yield of 34.4%. Additionally, no reduction occurred in the absence of the Cr(ii)PDTA or in the absence of Ni cyclam.

### Conventional homogeneous electrolysis

To our knowledge, conventional H-cell electrolysis with Ni cyclam has not been widely reported in exclusively aqueous media using a glassy carbon working electrode, with the lone example reporting 90% selectivity for CO in 0.1 M KCl electrolyte.^15^ Existing literature typically utilises either a primarily non-aqueous solvent or a mercury working electrode, both of which suppress hydrogen evolution at the electrode surface. Indeed, we did not observe significant CO_2_R activity by Ni cyclam in carbonate supporting electrolyte. Using applied potentials of −1.313 V and −1.373 V *vs.* Ag/AgCl, which roughly correspond to 50% and 90% Cr(ii)PDTA state of charge (SOC) respectively, we see almost exclusively H_2_ evolution under CO_2_R conditions (Table S3). While these potentials are slightly less negative than those commonly reported, they were chosen for fair comparison to the decoupled system we report herein. Notably, the presence of CrPDTA in the system drastically suppressed the charge passed, especially when both Ni cyclam and CrPDTA were present showing that parasitic HER at the electrode was suppressed by the mediator. Using the more commonly employed 0.1 M KCl the total charge passed over the experiment was much lower for all cases, and no appreciable CO_2_R activity was measured at −1.313 V or −1.373 V. Using a more reducing potential of −1.5 V resulted in a larger proportion of CO_2_R products (Table S4), however these were still much lower than previously reported.^15^

Employing Cr PDTA to enhance homogeneous H-cell electrolysis proved ineffective. Not only did we find little meaningful CO_2_R activity, but the combination of Cr PDTA and Ni cyclam also drastically reduced the background H_2_ such that very little charged was passed compared to control experiments. This is contrary to the behaviour we would expect from a booster, where the electron shuttling should extend the thickness of the diffusion-reaction layer and thus increase the observed current.^18^ Instead, we can conclude that the use of Cr PDTA as a reducing equivalent for batch decoupled electrolysis allows for CO_2_R activity of Ni cyclam under conditions (both potential and electrolyte) that would not allow for such activity in a conventional H-cell electrolyser. Even in 0.1 M KCl, no rate boosting behaviour was observed, however the presence of Cr PDTA did improve CO selectivity (but not productivity) by inhibiting H_2_ production. The corresponding controls highlighted a considerable difference in charge passed between 1 M KHCO_3_ and 0.1 M KCl, with the suppression of hydrogen evolution much less pronounced in 0.1 M KCl due to overall lower activity. As Cr PDTA is reduced at a potential slightly positive of Ni cyclam, we could expect very slow electron transfer from Cr to Ni, such that even with a 10× excess of mediator, no boosting was observed. Notably, we did not observe formate as a major product above background levels in any of the H-cell tests, highlighting the unique reactivity observed for the decoupled reaction.

### Mechanistic considerations

**Fig. 3:**
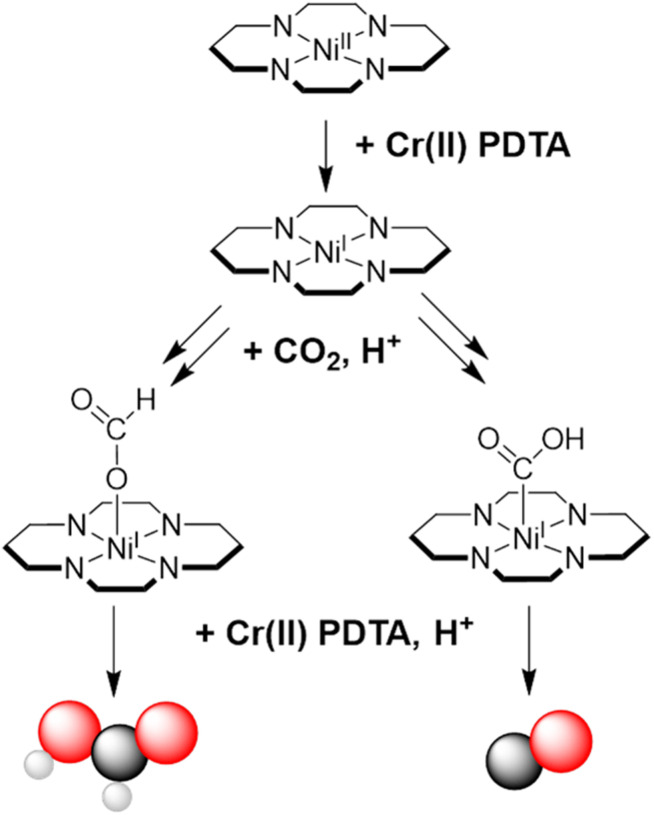
Suggested pathways towards the products CO and HCOO(H) based on existing literature, where CO_2_ binding mode directs product formation.^18^

Despite decades of study, CO_2_ reduction by Ni(cyclam) is not well understood mechanistically, and even recent years have witnessed fundamental changes to the mechanistic hypothesis.^19^ As mentioned, formate was not expected to be observed as a product, certainly not as a major product, although there is precedent for formate formation in DMF on mercury electrodes.^16^ Based on DFT studies in the literature, we attribute the different products to the formation of a carbon binding adduct for CO or an oxygen binding adduct for formate as shown in Fig. 3. Further, HER was not expected to contribute such a large portion of the products under CO_2_R conditions as it did with 1 M KCl. Previously this has been attributed to a low CO_2_ concentration,^20^ however these experiments were in a CO_2_ saturated solution and atmosphere. A report into the effects of using different pH 7 buffers in the supporting electrolyte during photochemical CO_2_R found that both activity and selectivity towards CO varied considerably between buffers.^21^ The authors proposed that both the size and charge of the buffer species resulted in changes to the secondary coordination sphere of the complex, promoting or demoting the reaction pathways to the various products. This is evident the from results presented herein, where using a bicarbonate buffered electrolyte promoted the selectivity of CO. Contrary to this, Sauvage previously reported poor performance in carbonate electrolyte, which they attributed to a high pH of 10.6, much higher than the pH 7.5 reported herein.^17^

It is important to note that electrolysis performed using Ni cyclam with a mercury cathode is considered to be a surface adsorbed process rather than truly homogeneous.^19,22^ Indeed, it is found that activity on a mercury electrode is much higher than any other material.^15^ Because there is no electrode surface onto which the catalyst can adsorb when used in the decoupled system, and we did not observe the formation of a surface coating within the reaction vessel, we assume that the reaction must be proceeding by a fully homogeneous process.

**Fig. 4:**
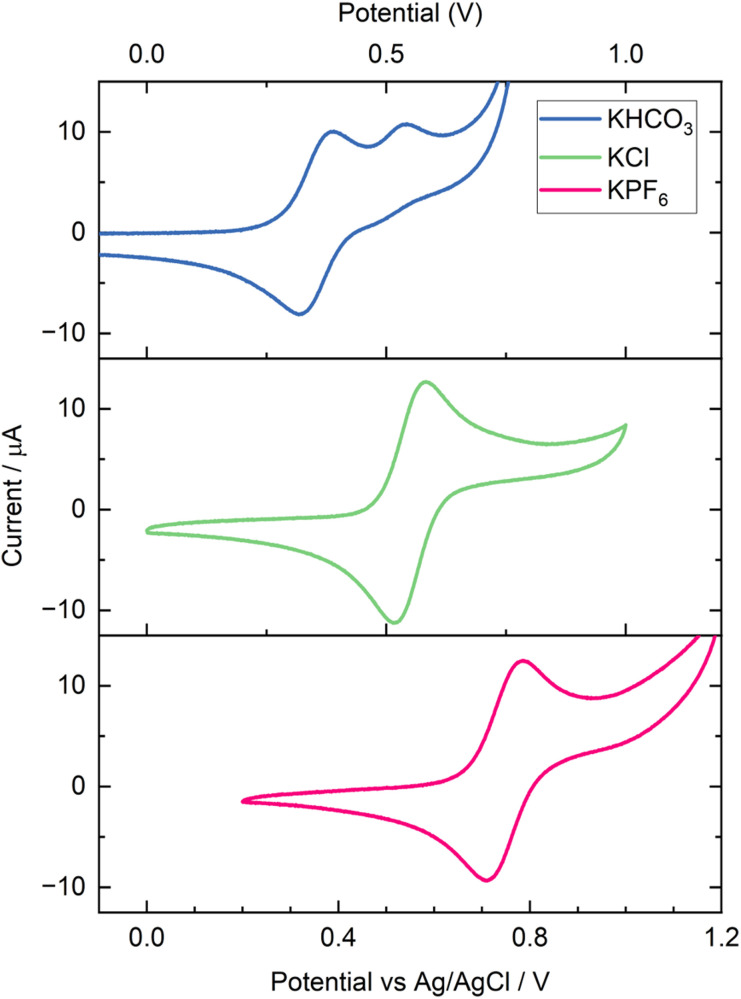
Response of the Ni(ii)/(iii) couple of Ni cyclam to a change in the supporting electrolyte anion. Cyclic voltammetry of 1 mM Ni cyclam Cl_2_ with 1 M KHCO_3_, 1 M KCl and 0.1 M KPF_6_ respectively, under N_2_ atmosphere.

The cyclam ligand itself is tetradentate, leaving an axial pair of coordination sites that may be occupied by a variety of species present in solution. Proposed mechanisms necessitate CO_2_ coordination in one of these sites, however the other site remains open to whatever species may be present in solution. No mechanistic study to date has considered this in determining the intermediates of the catalytic cycle. As prepared in solid form, the Ni cyclam complex contains two chloride anions as counter charge. Upon dissolution in water, these anions will undoubtedly exchange with other species in solution, most importantly CO_2_ when in its reduced Ni(i) form. Depending on what other anions are in solution, they may also exchange, as well as solvent molecules. In switching from an electrolyte dominated by KHCO_3_ to one dominated by KCl, it can be expected that the species occupying the vacant coordination site will change. Cyclic voltammetry of the non-catalytic Ni(ii)/Ni(iii) couple in KHCO_3_ was noticed to display a shoulder peak on the oxidation wave not evident when using KCl (Fig. 4). Additionally, the redox potential shifted more positive when saturated with CO_2_, which was not observed for KCl, despite the pH shift between N_2_ and CO_2_ being greater in the unbuffered KCl case (Fig. S7). CO_2_ saturation also resulted in a reduction in the magnitude of the shoulder peak. A further electrolyte was tested, 0.1 M KPF_6_, with the intention of it being poorly coordinating, which resulted in clean voltammetry free of any shoulder peaks. Upon closer inspection, it was noted that the potential of this couple shifts based on supporting electrolyte, in the order KHCO_3_ (N_2_) < KHCO_3_ (CO_2_) < KCl < KPF_6_ (Fig. 4).

UV-vis spectra of Ni cyclam with KCl and KHCO_3_ electrolytes (Fig. S11–S13) highlighted a subtle difference in electronic structure. Increasing the concentration of Ni cyclam to 10 mM to increase absorbance, a slight reduction in intensity of the yellow-brown colour was observed for the KHCO_3_ sample compared to KCl. This was confirmed spectroscopically, with a slight blueshift of the peak from 455 to 450 nm and a 20% drop in absorbance. More importantly, a second peak at ∼340 nm became more intense, which increased further under CO_2_ saturation, with a corresponding drop in main peak intensity. Most surprisingly, after a few days the KHCO_3_ solution appeared to have aged and now displayed pale purple colouration, which was not typically observed. UV-vis of this solution highlighted a considerable drop in the ∼450 nm peak intensity, and corresponding increase for the ∼340 nm peak, along with a new broad shoulder at ∼550 nm. Cyclic voltammetry also displayed a much larger shoulder for this aged solution for the first few scans, before settling into the expected response, suggesting that oxidation to Ni(iii) restores the complex to its original state (Fig. S8). Solution state IR was unable to observe any peaks corresponding to Ni cyclam ligand interactions and thus could not be used to determine a change in coordination environment. This aging was not typically observed, with much older samples not exhibiting any change.

A further series of CVs were conducted in 1 M KCl to determine the effect of pH on the Ni(ii)/(iii) couple, shown in Fig. S9. Initially, a small amount of HCl was added dropwise, lowering the pH to 3, after which CVs were taken at integer values up to pH 14 by dropwise addition of KOH solution after each scan. The total volume was increased by a factor of 2.5× by the end of the experiment, with the dilution primarily contributing to the initial decrease in peak size as the pH increased. Between pH 3–10, minimal change in peak symmetry and position was observed, as expected for a non-proton-coupled redox process. Above this pH however, reversibility was initially lost, with no corresponding reduction back to Ni(ii) after oxidation at pH 12. The reduction peak returned as pH was increased further, and at pH 14 the voltammetry appeared consistent with that of Ni(OH)_2_/Ni(OOH) redox under alkaline conditions. This final pH step was accompanied by a shift in colour toward that of the aged 1 M KHCO_3_ solution.

Hypothesising that the formation of CO_3_^2−^ may be the cause of the colour change with time and pH, a 1 M K_2_CO_3_ electrolyte was evaluated. This did result in a purple solution, which appeared much like the Ni cyclam Cl_2_ in its solid form. The corresponding voltammetry (Fig. S10) was poor, giving a more exaggerated form of the aged 1 M KHCO_3_ solution, with very low reversibility and an even larger shoulder. This strong colour change does not appear to be the result of pH alone, as the pH was around 12.2, while full purple colouration in the absence of carbonate did not occur even above pH 14. Furthermore, while poor, the voltammetry did at least display some reversibility, which had already been lost at pH 12 in the KCl/KOH electrolyte.

This colour change is especially interesting, as to our knowledge it has not been previously reported. Indeed, even the photolysis study in a variety of buffers made no mention of colour changes or differences in UV-vis spectra between electrolytes, something that is critical when reaction depends on the absorption of incident light.^21^ The study did not observe shoulder peaks in the Ni(ii)/Ni(iii) redox in any of the buffers tested, contrary to our own observations for carbonate containing electrolytes.

Clearly, there is some change in coordination environment when carbonate is present, and it is highly likely this contributes to the effects observed on the catalytic cycle and turnover. This, however, does not seem to result in promoting formate selectivity in conventional electrolysis, only in the decoupled system. It is possible this difference is caused by the fact that conventional Ni cyclam CO_2_ reduction is considered a surface adsorbed process, and as such there is no co-ligand in the catalytic cycle. However, as DECO_2_R is occurring entirely in solution, the process is open to the influence of co-ligand effects, resulting in broad changes in product selectivity between electrolytes.

Beyond coordination effects, we may also consider the availability of protons in the reaction between different electrolytes. However, the typical assumption that increased proton availability would lead to increased production of pathways that consume more protons does not correlate with our observations. The production of both H_2_ and CO require two protonation events, however formate strictly only requires one if the product is taken as potassium formate rather than formic acid. However, we observe that as the proportion of KHCO_3_ is reduced and CO selectivity goes down, H_2_ and formate selectivity go up. On one hand, decreasing KHCO_3_ concentration will lower the pH, as the buffering capacity will be reduced, however HCO_3_^−^ has a p*K*_a_ of 10.33, much lower than water, so is a more readily available proton source when present.

From the DECO_2_R results, we see that CO is produced much more selectively when a large amount of KHCO_3_ is present, however H_2_ production is suppressed under the same conditions. Furthermore, from the H-cell study, we see that HER is much more prevalent in KHCO_3_ electrolyte than KCl electrolyte for conventional electrolysis. The contrary nature of these results makes determining the full effect of each component on the observed reactivity difficult to deconvolute.

### Scaled DECO_2_R and preliminary kinetics

A preliminary test employing Ni cyclam as the catalyst in an online analysis flow reactor was performed with increased mediator concentration compared to the batch tests. Using the same volumes as in the stationary experiment, but with 0.1 M Cr PDTA mediator to provide enough electrons for 50 catalyst turnovers, a continuous flow of CO_2_ at 10 mL min^−1^ was bubbled through the reactor and into the injection loop of the gas chromatograph, where the composition of the gas was analysed every 20 minutes.

**Fig. 5:**
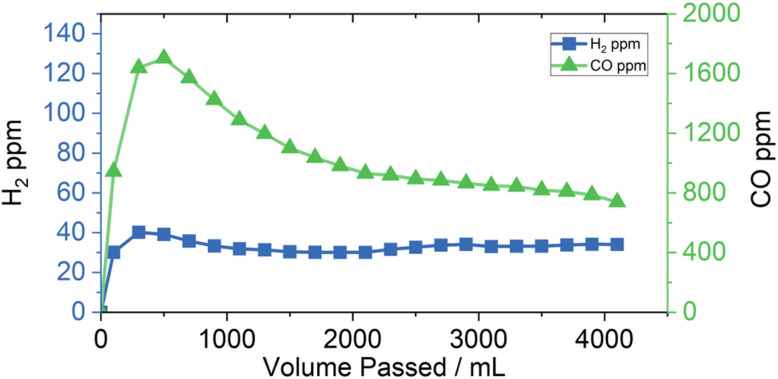
Graph of the H_2_ and CO concentration in the outflowing gas from the online reactor as a function of time and volume passed. The electrolyte consisted of 100 mM Cr PDTA and 1 mM Ni cyclam with 1 M KHCO_3_, with a CO_2_ flow of 10 mL min^−1^.

Fig. 5 shows how the concentration of products in the outflowing gas evolved over time. The concentration of both CO and H_2_ increased over the first hour of electrolysis, peaking at 1700 and 40 ppm respectively. H_2_ concentration remained between 30 and 40 ppm for the following 6 hours of online analysis, while the CO concentration initially decayed rapidly to 1300 ppm after just one more hour, and to 740 ppm after the full 7 hours. The total amount of CO and H_2_ produced over the 7 hours was 4.32 and 0.134 mL (180 and 5.58 µmol) respectively. This can be approximated to a peak production rate of 17.0 μL (0.708 nmol) of CO per minute, or an average of 10.5 μL (0.438 nmol) per minute for the full 7 hours. The final composition of the headspace of the flask was found to contain an additional 1.04 and 0.0744 mL (43 and 3.1 µmol) of CO and H_2_ respectively, for total product yields of 5.36 and 0.208 mL (223 and 8.7 µmol). This can be equated to faradaic yields of 22.3% and 0.87%, with a further 5.75% (57.5 µmol) yield of HCOO(H) in the electrolyte, corresponding to selectivities of 3%, 77%, and 20% for H_2_, CO, and HCOOH, which matches the results obtained from the lower concentration batch reaction. The FY however was much poorer, at only 29% compared to the 51% observed at the lower concentration. This would give a final turnover number of 14.5. Turnover frequency towards CO over the first 7 hours was approximately 1.3 h^−1^, comparable to that reported by Sauvage at low overpotential.^17^ Because the catalytic cyclic relies on two electron transfer events, one before CO_2_ complexation and one after, and the Cr PDTA mediator reduction potential falls slightly positive of Ni cyclam thus disfavouring electron transfer, it is likely that the turnover of the process will be very low. It will continue to slow considerably as the SOC of the mediator is depleted and the rate of electron transfer decreases due to increase in potential gap between the mediator and the catalyst, akin to how rate decreases with lowered overpotential in a conventional system.

It has been suggested that the rate of catalytic turnover for Ni cyclam is limited by the formation of nickel carbonyl species that deactivate the catalyst.^23^ The observed faradaic yield of just 29% may indicate that the catalyst is being deactivated before it can complete the 50 turnovers, however there was no visible deterioration of the electrolyte.

An initial exploration into the kinetics of the reaction was performed using UV-vis spectrometry to measure how the ratio of Cr(ii)/Cr(iii) changes over time. The green charged state has an absorption peak at 666 nm with a weak extinction coefficient of 12.4 L mol^−1^ cm^−1^, compared to the more strongly absorbing peak for the red discharged state at 506 nm with an extinction coefficient of 116 L mol^−1^ cm^−1^. This strongly absorbing peak can thus be used as an indicator of Cr(iii) concentration with minimal interference from Cr(ii), which can be used to determine the state of charge of the mediator solution through a linear relationship.

**Fig. 6:**
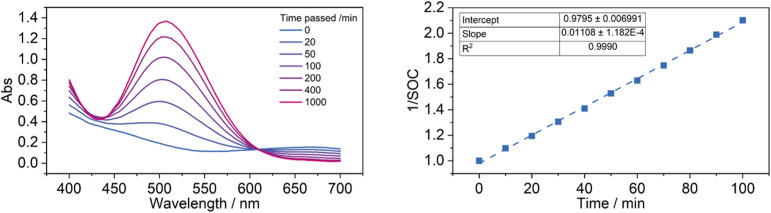
(Left) Spectra of the mediator-catalyst mixture over time, showing increase in absorption at 506 nm as the concentration of Cr(ii) falls and the concentration of Cr(iii) increases. (Right) Plot of the reciprocal of SOC against time for the first 100 minutes of reaction. SOC was determined from relative absorption assuming 100% at time = 0 min and 0% at time = 1000 min from a linear Beer–Lambert association.

A solution of 10 mM Cr(ii) PDTA and 1 mM Ni cyclam was prepared and rapidly added to a sealed quartz cuvette under CO_2_ atmosphere, where the progress of the reaction was measured by spectrometry between 400–700 nm every 10 minutes. 100 spectra were obtained, highlights of which are shown in Fig. 6. Initially, the concentration of Cr(ii) fell rapidly, reaching 48% state of charge (SOC) after 100 minutes of reaction. Analysis of this initial rate indicates that the reaction is second order with respect to Cr(ii) concentration, with the plot of 1/SOC *vs.* time being most linear with an *R*^2^ of 0.9990, compared to the *R*^2^ value of 0.9947 for the plot of ln(SOC) *vs.* time.

Because we are unsure if/how much the Ni cyclam catalyst is degrading over the course of the experiment, it is not possible to know whether this instead is the reason that the rate slowed considerably as the reaction proceeded, and the reaction may not necessarily be second order with respect to Cr(ii) concentration. While the excellent potential overlap shown in Fig. 1 enables minimal loss of energy efficiency by using the RM on cell voltage, there is no thermodynamic drive for the mediator to exclusively reduce the catalyst and instead they will exist in equilibrium at a shared solution potential. It can be expected that as the state of charge of the mediator is depleted, a lesser proportion of the catalyst exists in the reduced state which is active towards CO_2_R, further slowing the rate of turnover.

## Experimental

### Chemicals

All chemicals were used as purchased without further purification. Sodium chloride (99.5%) potassium chloride (99.5%), potassium hydrogen carbonate (99.7%), nickel chloride hexahydrate (98%), potassium ferrocyanide (99%), ethanol (absolute), acetone (99%), and diethyl ether (99%) were purchased from Thermo Fisher (Alfa Aesar, Acros Organics).

Chromium potassium sulphate dodecahydrate, 1,3-propanediamine-*N*,*N*,*N*′,*N*′-tetraacetic acid (99%), 1,4,8,11-tetraazacyclotetradecane (cyclam, 98%), and potassium hydroxide (reagent grade) were purchased from Merck (Sigma Aldrich). CO_2_ gas was purchased from BOC Ltd. Isopropanol (99.5%) was purchased from Honeywell.

### Synthetic procedures

KCrPDTA was synthesised following previously reported procedure.^14,24^ KCr(SO_4_)_2_·12H_2_O (29.6 g, 59.3 mmol) was dissolved in water (120 mL) alongside H_4_-PDTA (18.4 g, 60.1 mmol), and the resulting purple solution was refluxed at 100 °C for 4 hours. The resulting red solution was partially neutralised by the addition of KOH flakes (12 g), and was refluxed for a further 18 hours. The solution was then removed from the heat and fully neutralised by dropwise addition of 1 M KOH. To this, an equal volume of acetone (around 200 mL) was added, resulting in immediate formation of an off-white precipitate, K_2_SO_4_, which was removed by vacuum filtration. The Volume of the filtrate was then reduced to around 60 mL by rotary evaporation, after which it was added slowly to ice cold isopropanol (350 mL), resulting in a dark red microcrystalline precipitate. This was collected by vacuum filtration and dried at 70 °C. The isolated crystals are KCrPDTA·3H_2_O, M.W. 447 g mol^−1^.

Ni(cyclam)Cl_2_ was synthesised by a rapid reaction of the ligand with nickel chloride.^25^ NiCl_2_·6H_2_O (1.16 g) was dissolved in ethanol (80 mL) heated to 50 °C. To this, cyclam (1.0 g) was added resulting in a rapid colour charge from green to pale purple. Once cooled, diethyl ether (20 mL) was added resulting in immediate precipitation of a pale purple solid, which was collected by filtration and dried under vacuum for a quantitative yield.

### Electrochemical methods

Electrochemical experiments were performed using either a Biologic SP300 or Ivium Vertex potentiostat. Cyclic voltammetry was performed in a small glass cell of approximately 20 mL volume, using a 3 mm diameter glassy carbon working electrode unless otherwise stated. For aqueous experiments, the reference electrode was typically Ag/AgCl, using 3 M NaCl inner solution. The potentials of the reference electrodes were periodically checked against a saturated calomel reference electrode to compensate for any potential drift over time, and all potentials reported against RHE throughout the work were calculated related to this.

To access the charged state of the mediator, a large electrochemical flow cell with geometric electrode areas of 16 cm^2^ was used to charge the electrolytes for decoupled CO_2_ reduction and to test performance of the electrolytes as a cell. The cell was constructed from an outer frame of steel, polypropylene electrolyte diffusers, brass current collectors, graphite composite electrode plates, graphite felt electrodes, EPDM gaskets, and Fumatek Fumapem F-930 cation exchange membrane. The Cr PDTA negolyte was charged against K_4_Fe(CN)_6_ posolyte as a sacrificial electron source. It is further described in the SI, Fig. S16.

### Batch DECO_2_R

The initial results were obtained from a batch reaction performed in a Schlenk flask. Air was removed from the flask by alternating between vacuum (<1 mBar) and CO_2_ (or N_2_ in the case of controls) 5× to eliminate as much oxygen a reasonably possible. To this, catalyst and mediator solutions saturated with CO_2_ (or N_2_) were injected through a PTFE faced silicone septum. The resulting CO_2_R products were analysed by a combination of gas and ion chromatography.

### DECO_2_R with online GC

As an initial design, a three necked round bottom flask was used as a makeshift flow reactor. In this instance, only the gas was flowing. CO_2_ was injected through one arm of the flask by a needle submerged below the electrolyte surface. A second needle in the other arm allowed headspace gas to flow out of the flask and through the injection loop of the GC where its composition was analysed every 20 minutes. Mediator and catalyst were added by the same manner as described for the batch reactor design. This allowed for the collection of semi-quantitative rate data, as the composition of the outflowing gas reflected the rate of production. The volume of the flask was measured to be 163.15 cm^3^.

### H-cell electrolysis

H-cell electrolysis was performed in a custom glass H-cell (Fig. S17). The volume of the working compartment is approximately 87 mL, of which 7 mL is taken up by electrodes, 40 mL by electrolyte, and 40 mL by gas headspace. The working compartment was separated from the counter by a Fumapem F-930 cation exchange membrane. The working electrode consisted of a glassy carbon plate with a confined area of 1.2 × 1.2 cm, for 1.44 cm^2^. The connecting copper wire is plastic coated to avoid contamination.

### Product quantification

Calibrations were prepared to allow for the quantification of the products H_2_, CO, CH_4_, and C_2_H_4_. The gases were analysed on a Shimadzu 2030 GC system equipped with a ResTek ShinCarbon ST 80/100 column and barrier ionisation discharge (BID) detector, using helium as the eluent gas. Linear calibrations in the range 200–1000 ppm were prepared for all four gases, with further curved calibrations in the range 2 000–100 000 prepared for H_2_ and CO. Certified calibration gas standards were purchased from BOC Ltd. The range of concentrations were prepared by dilution of these standards with CO_2_ by the use of two gas tight syringes. Calibration data can be found in the SI.

## Conclusion

In summary, a brief exploration into using Ni cyclam as a homogeneous catalyst for DECO_2_R with Cr PDTA as a RM has been demonstrated. The high selectivity of the molecular catalyst resulted in good selectivity towards ECO_2_R reported in this work, with hydrogen evolution accounting for just 3–5% of the observed products when using KHCO_3_ as the supporting electrolyte. Unfortunately, this was coupled with a relatively low faradaic yield ranging from 50–75%. An interesting observation was found in that using KCl as the main supporting electrolyte resulted in a switch in selectivity from CO to HCOOH, which is not typically seen as a major product using Ni cyclam under aqueous conditions. Scaling up the reaction to increase the total charge passed saw a further decrease in the faradaic yield, suggesting that the catalyst may be deactivated by side reactions during DECO_2_R.

Further characterisation of the complex in a range of supporting electrolytes, primarily focused on the non-catalytic Ni(ii)/Ni(iii) redox couple, highlighted a change in the coordination environment. While some difference in activity between electrolytes has previously been reported, no attention was paid to the coordination environment of the complex. Indeed, as the mechanism is considered surface adsorbed in conventional electrolysis, coordination in solution is not likely to have much influence on the mechanistic cycle. In the decoupled system reported herein however, it appears that electrolyte coordination has a significant impact on the catalytic cycle of this fully homogeneous process.

## Conflicts of interest

There are no conflicts to declare.

## Supplementary Material

DT-055-D5DT02738A-s001

## Data Availability

Data for this article, including raw data files and figure processing and data analysis software files are available at Zenodo at DOI: https://doi.org/10.5281/zenodo.16752251. Some data supporting this article have been included as part of the supplementary information (SI). Supplementary information is available. See DOI: https://doi.org/10.1039/d5dt02738a.
