# Supplementary material for: Homogeneous redox catalysed reduction of CO2 by nickel cyclam catalyst and chromium-based redox mediator
Source: Dalton Trans. 2026 Apr 24;55(18):7365–72. doi: 10.1039/d5dt02738a (PMC13112525; doi:10.1039/d5dt02738a)
Supplement: DT-055-D5DT02738A-s001 [file DT-055-D5DT02738A-s001.pdf]

Supplementary Information (SI) for Dalton Transactions.
This journal is © The Royal Society of Chemistry 2026

Homogeneous Redox Catalysed Reduction of CO_2 by Nickel Cyclam Catalyst and Chromium-Based Redox Mediator

Mark Potter, Kathryn Toghill

Additional data

1. **Voltammetry of the Ni (I)/(II) cyclam couple and Cr PDTA.**

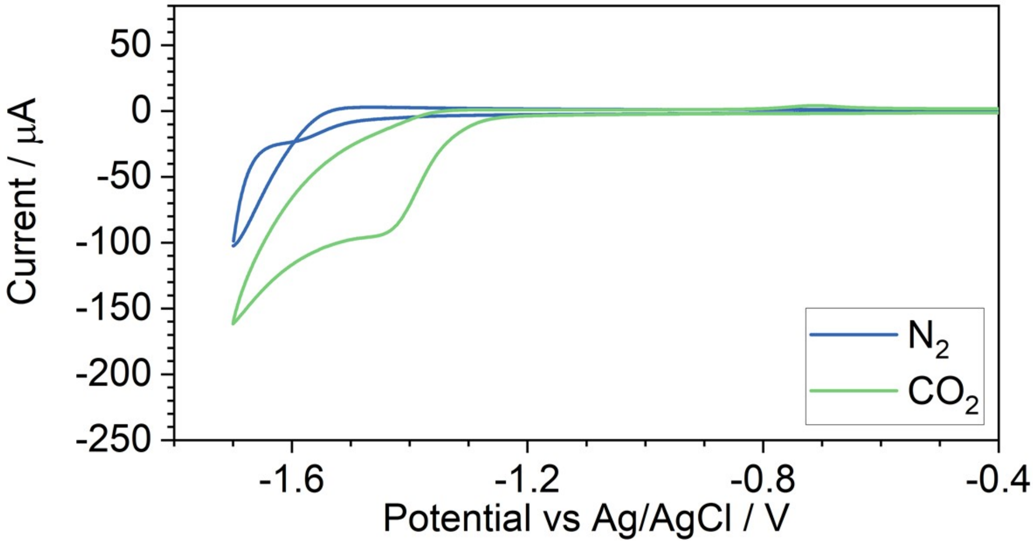

Figure S1. *Cyclic voltammetry of* ***1 mM Ni cyclam Cl_2*** *with* ***1 M KCl*** *in water under* ***N_2*** *(blue) and* ***CO_2*** *(green) atmospheres, using a glassy carbon working electrode at a scan rate of 100 mVs^{-1}. Catalytic current is evident under CO_2.*

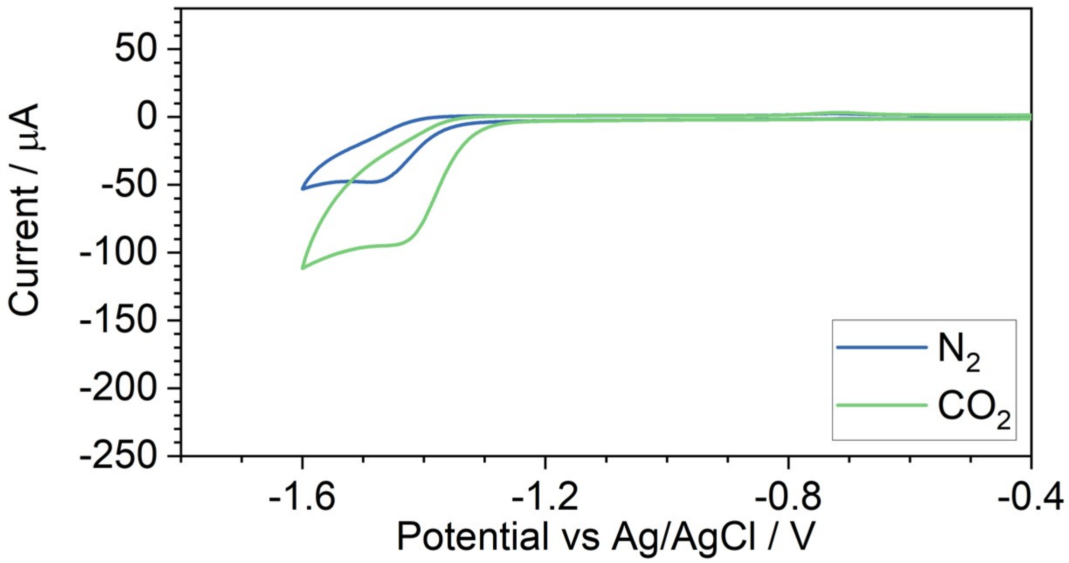

Figure S2. *Cyclic voltammetry of **1 mM Ni cyclam Cl$_2$** with **1 M KHCO$_3$** in water under **N$_2$** (blue) and **CO$_2$** (green) atmospheres, using a glassy carbon working electrode at a scan rate of 100 mVs^{-1}. Catalytic current is evident in both regimes; however, it is much larger under CO$_2$.*

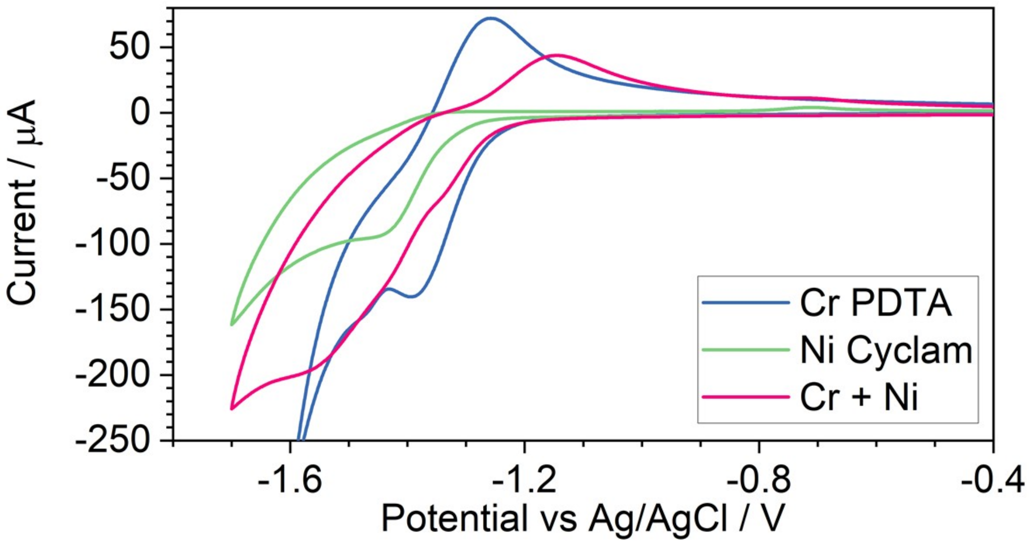

Figure S3. *Cyclic voltammetry of **10 mM Cr PDTA** (blue), **1 mM Ni cyclam Cl$_2$** (green), and **10 mM Cr PDTA + 1 mM Ni cyclam Cl$_2$** (pink) with **1 M KCl** in water under **CO$_2$** atmosphere, using a glassy carbon working electrode at a scan rate of 100 mVs^{-1}. pH 5.5*

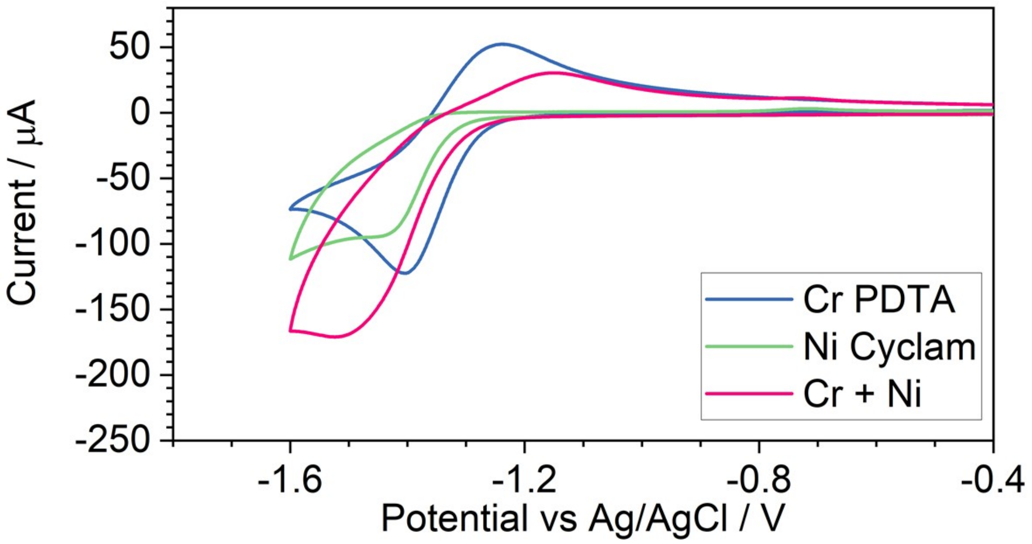

Figure S4. *Cyclic voltammetry of **10 mM Cr PDTA** (blue), **1 mM Ni cyclam Cl_2** (green), and **10 mM Cr PDTA + 1 mM Ni cyclam Cl_2** (pink) with **1 M $KHCO_3$** in water under **CO_2** atmosphere, using a glassy carbon working electrode at a scan rate of 100 mVs^{-1}. pH 7*

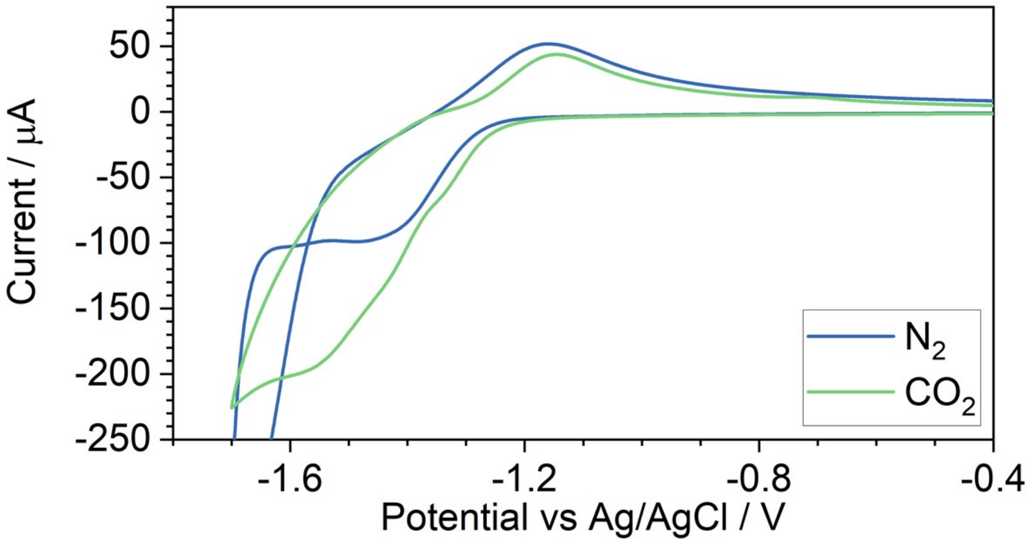

Figure S5. *Cyclic voltammetry of **10 mM Cr PDTA** + **1 mM Ni cyclam Cl_2** with **1 M KCl** in water under **N_2** (blue) and **CO_2** (green) atmospheres, using a glassy carbon working electrode at a scan rate of 100 mVs^{-1}.*

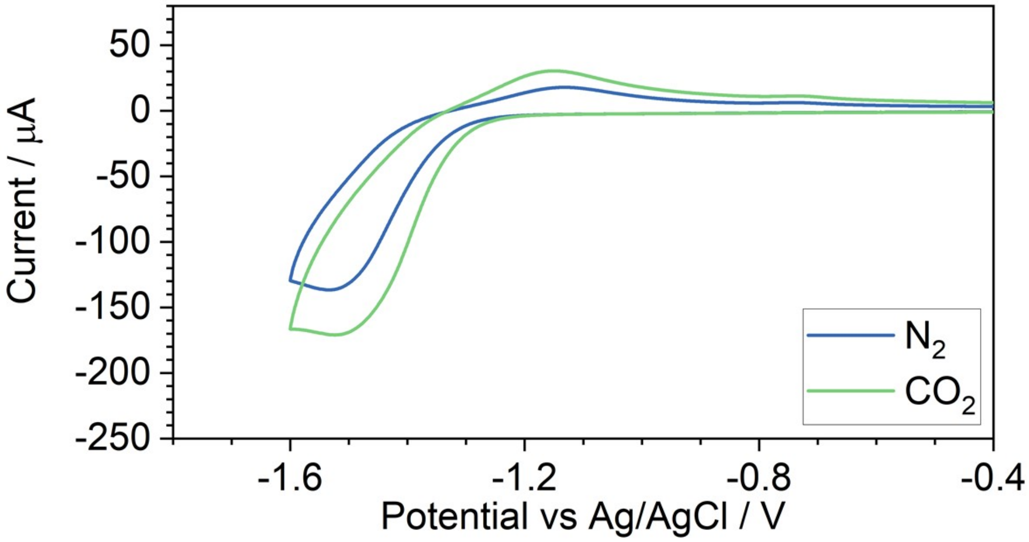

Figure S6. *Cyclic voltammetry of **10 mM Cr PDTA + 1 mM Ni cyclam Cl_2** with 1 M **$KHCO_3$** in water under **N_2** (blue) and **CO_2** (green) atmospheres, using a glassy carbon working electrode at a scan rate of 100 mVs^{-1}.*

2. Electrolysis of CO_2 using Ni cyclam

Results

Table S1. *Full table of faradaic yields for DECO$_2$R using Ni cyclam catalyst with Cr PDTA mediator, with σ indicating the standard deviation within each set, as plotted in* ***Figure 2.***

Faradaic Yields	H_2	σ	CO	σ	HCOO(H)	σ	Total	σ
1 M $KHCO_3$	1.41		40.07		10.45		51.93	
	1.14		36.44		9.72		47.3	
	1.28		42.47		10.95		54.7	
Average	1.28	0.11	39.66	2.48	10.37	0.51	51.31	3.05
0.9 M $KHCO_3$ 0.1 M KCl	1.22		48.05		11.57		60.84	
	1.17		48.20		11.18		60.55	
	1.29		51.82		12.06		65.17	
Average	1.23	0.05	49.36	1.74	11.60	0.36	62.19	2.11
0.5M KCl 0.5M $KHCO_3$	2.1		45.32		19.57		66.99	
	1.85		45.7		18.68		66.23	
	1.87		44.9		21.53		68.3	
Average	1.94	0.11	45.31	0.33	19.93	1.19	67.17	0.85
0.1 M $KHCO_3$ 0.9 M KCl	3.89		31.19		39.52		74.6	
	4.07		29.98		36.56		70.61	
	3.70		31.44		38.36		73.5	
Average	3.89	0.15	30.87	0.64	38.15	1.22	72.90	1.68
1 M KCl	13.72		21.39		39.05		74.16	
	13.56		22.95		37.34		73.85	
	15.75		22.06		38.26		76.07	
Average	14.34	1.00	22.13	0.64	38.22	0.70	74.69	0.98
1 M KCl N_2	34.44							
	33.41							
	35.32							
Average	34.39	0.78						

Table S2. *Full table of relative yields for $DECO_2R$ using Ni cyclam catalyst with Cr PDTA mediator, with σ indicating the standard deviation within each set, as plotted in **Figure 2.***

Relative Yields	H_2	σ	CO	σ	HCOO(H)	σ
1 M $KHCO_3$	2.72%		77.16%		20.12%	
	2.41%		77.04%		20.55%	
	2.34%		77.64%		20.02%	
Average	2.49%	0.16%	77.28%	0.26%	20.23%	0.23%
0.9 M $KHCO_3$ 0.1 M KCl	2.01%		78.98%		19.02%	
	1.93%		79.60%		18.46%	
	1.98%		79.52%		18.51%	
Average	1.97%	0.03%	79.37%	0.28%	18.66%	0.25%
0.5M KCl 0.5M $KHCO_3$	3.13%		67.65%		29.21%	
	2.79%		69.00%		28.20%	
	2.74%		65.74%		31.52%	
Average	2.89%	0.18%	67.46%	1.34%	29.65%	1.39%
0.1 M $KHCO_3$ 0.9 M KCl	5.21%		41.81%		52.98%	
	5.76%		42.46%		51.78%	
	5.03%		42.78%		52.19%	
Average	5.34%	0.31%	42.35%	0.40%	52.31%	0.50%
1 M KCl	18.50%		28.84%		52.66%	
	18.36%		31.08%		50.56%	
	20.70%		29.00%		50.30%	
Average	19.19%	1.07%	29.64%	1.02%	51.17%	1.06%

Table S3. *Faradaic yields of conventional H-cell electrolysis of CO_2 using* ***1 mM Ni cyclam Cl_2 catalyst and 10 mM Cr PDTA*** *in* ***1 M $KHCO_3$*** *supporting electrolyte. Bulk electrolysis was performed for 1 hour at the indicated potential in a glass H-cell using a 1.44 cm^2 glassy carbon plate working electrode, Ag/AgCl (3 M NaCl) reference electrode and platinum counter electrode, with the working and counter compartment separated by a Fumatek FS-930 cation exchange membrane.*

		Faradaic Yields % / absolute (μmol)			
-1.313 (V)	Charge passed (C)	H_2	CO	HCOOH	Total
Ni cyclam (1 mM)	24.5	80 (102)	trace	trace	80 (102)
Ni cyclam (1 mM) + Cr PDTA (10 mM)	1.4	40 (3.01)	2 (0.125)	23 (1.73)	65 (4.86)
$KHCO_3$ Ctrl	22.9	65 (77.1)	0 (0)	0 (0)	65 (77.1)
10 mM Cr PDTA ctrl	6.6	49 (16.9)	trace	1.9 (0.652)	51 (17.5)

-1.373 (V)	Charge passed (C)	H_2	CO	HCOOH	Total
Ni cyclam (1 mM)	39.5	81 (166)	trace	trace	81 (166)
Ni cyclam (1 mM) + Cr PDTA (10 mM)	0.7	80 (2.93)	0 (0)	24 (0.871)	104 (3.80)
$KHCO_3$ Ctrl	43.9	79 (181)	0 (0)	trace	79 (181)
10 mM Cr PDTA ctrl	9.4	65 (31.7)	trace	1 (0.711)	67 (32.4)

Table S4. *Faradaic yields of conventional H-cell electrolysis of CO_2 using* ***1 mM Ni Cyclam Cl_2 catalyst and 10 mM Cr PDTA*** *in* ***0.1 M KCl*** *supporting electrolyte. Bulk electrolysis was performed for 1 hour at the indicated potential in a glass H-cell using a 1.44 cm^2 glassy carbon plate working electrode, Ag/AgCl (3 M NaCl) reference electrode and platinum counter electrode, with the working and counter compartment separated by a Fumatek FS-930 cation exchange membrane. Potentials of -1.313 V, -1.373 V and -1.5 V represent 50% state of charge of Cr PDTA, 90% SOC, and literature electrolysis potential.*

		Faradaic Yields % / absolute (μmol)			
-1.313 (V)	Charge passed (C)	H_2	CO	HCOOH	Total
Ni cyclam (1 mM)	6.9	64 (22.9)	0 (0)	1 (0.505)	66 (23.4)
Ni cyclam (1 mM) + Cr PDTA (10 mM)	1.3	37 (2.42)	2 (0.139)	trace	39 (2.56)
0.1 M KCl ctrl	1.1	47 (2.79)	0 (0)	9 (0.530)	56 (3.32)
10 mM Cr PDTA ctrl	1.4	46 (3.37)	0 (0)	trace	46 (3.37)

-1.373 (V)	Charge passed (C)	H_2	CO	HCOOH	Total
Ni cyclam (1 mM)	3.0	35 (5.37)	5 (0.842)	3 (0.495)	43 (6.70)
Ni cyclam (1 mM) + Cr PDTA (10 mM)	1.6	24 (1.95)	12 (1.00)	trace	36 (2.95)
0.1 M KCl ctrl	1.4	53 (3.74)	0 (0)	trace	53 (3.74)
10 mM Cr PDTA ctrl	1.7	44 (3.88)	0 (0)	trace	44 (3.88)

-1.5 (V)	Charge passed (C)	H_2	CO	HCOOH	Total
Ni cyclam (1 mM)	7.7	30 (12.0)	18 (7.04)	2 (0.652)	49 (19.7)
Ni cyclam (1 mM) + Cr PDTA (10 mM)	3.5	7 .3(1.33)	33 (6.06)	trace	41 (7.39)
0.1 M KCl ctrl	1.6	45 (3.84)	0.3 (0.026)	6 (0.490)	51 (4.36)
10 mM Cr PDTA ctrl	3.2	32 (5.31)	0.2 (0.033)	3 (0.490)	35 (5.84)

Voltammetry of the Ni(II)/(III) redox couple

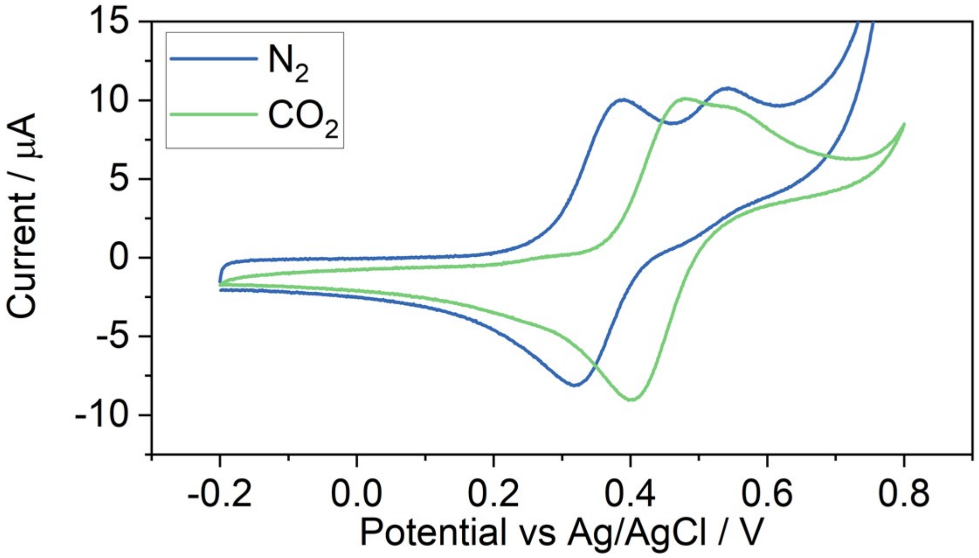

Figure S7. *Cyclic voltammetry of fresh* ***1 mM Ni cyclam Cl*** $_2$ *with* ***1 M KHCO*** $_3$ *under* ***N*** $_2$ *and* ***CO*** $_2$ *atmospheres.*

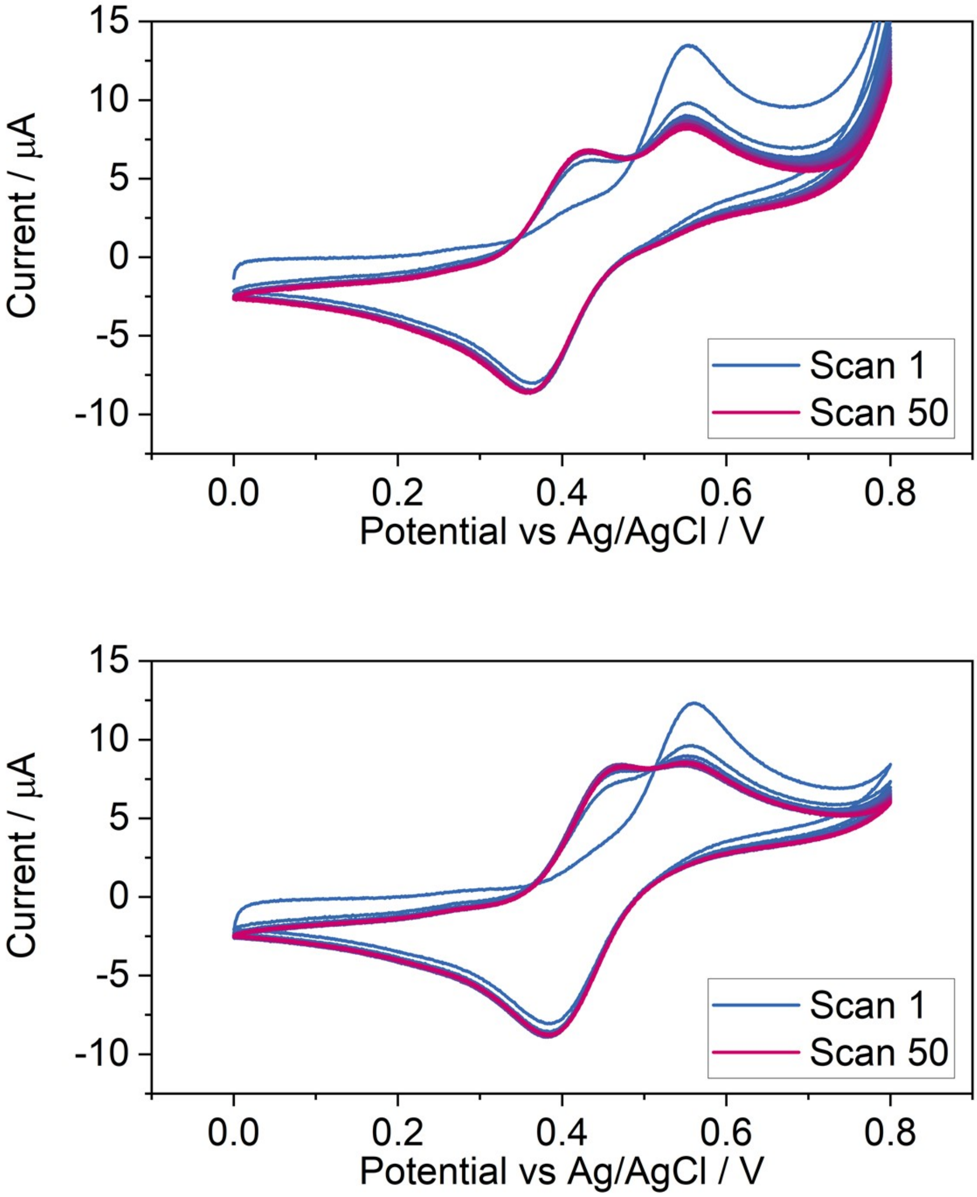

Figure S8. *Cyclic voltammetry of aged **1 mM Ni cyclam** $\mathbf{Cl_2}$ with **1 M** $\mathbf{KHCO_3}$ under* $\mathbf{N_2}$ *(top) and* $\mathbf{CO_2}$ *(bottom).*

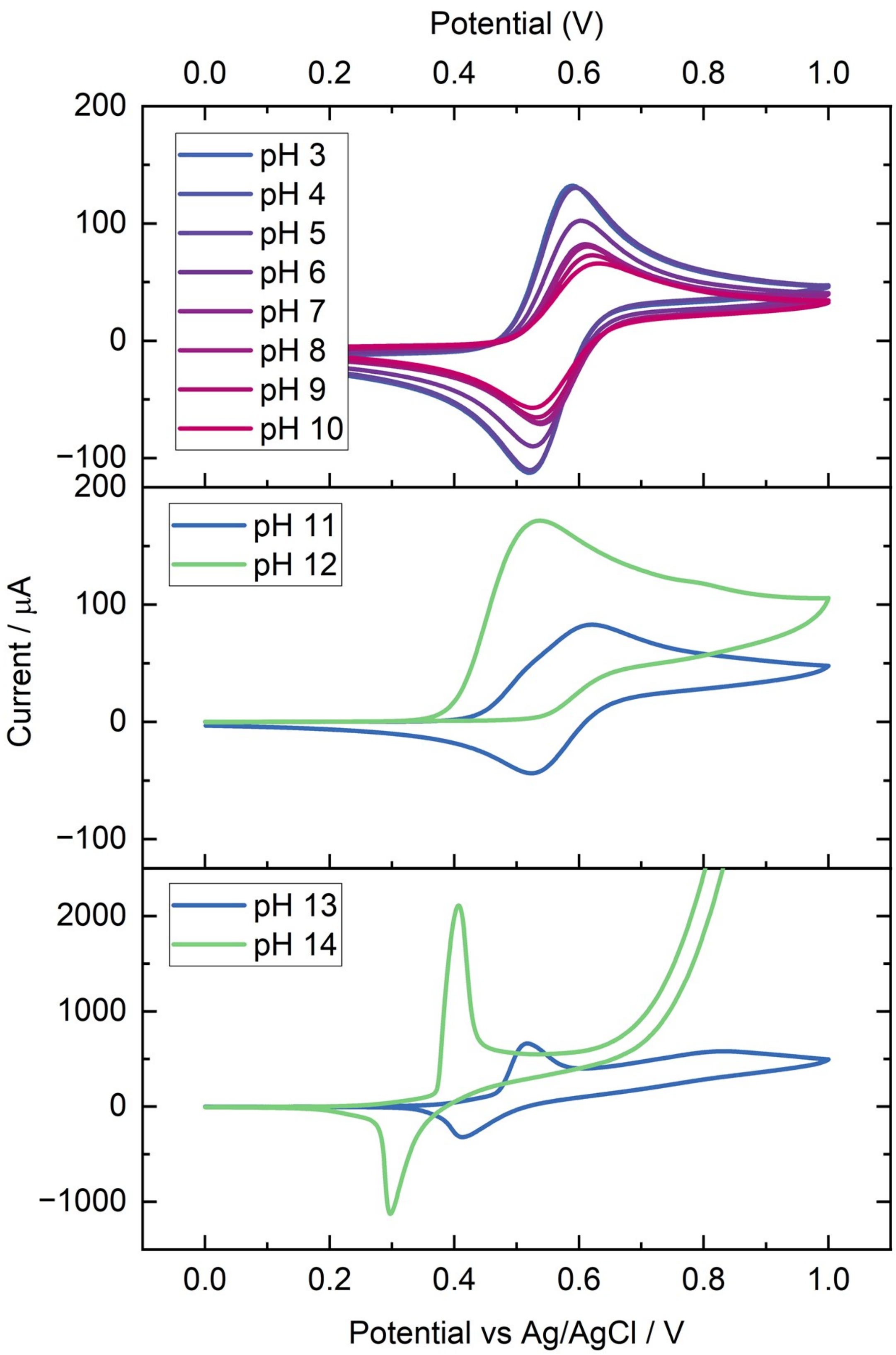

Figure S9. *Cyclic voltammetry of* ***Ni cyclam Cl$_2$*** *with* ***1 M KCl*** *under* ***air*** *atmosphere. The pH was initially adjusted to 3 by the addition of HCl solution, after which KOH and HCl solution were carefully added to increase the pH by approximately integer increments. The initial volume of electrolyte was 10mL, and the final volume was around 25 mL. As such, the Ni concentration decreased for each*

subsequent voltammogram, and the initial decrease in peak current from pH 3-10 was caused at least partially by dilution.

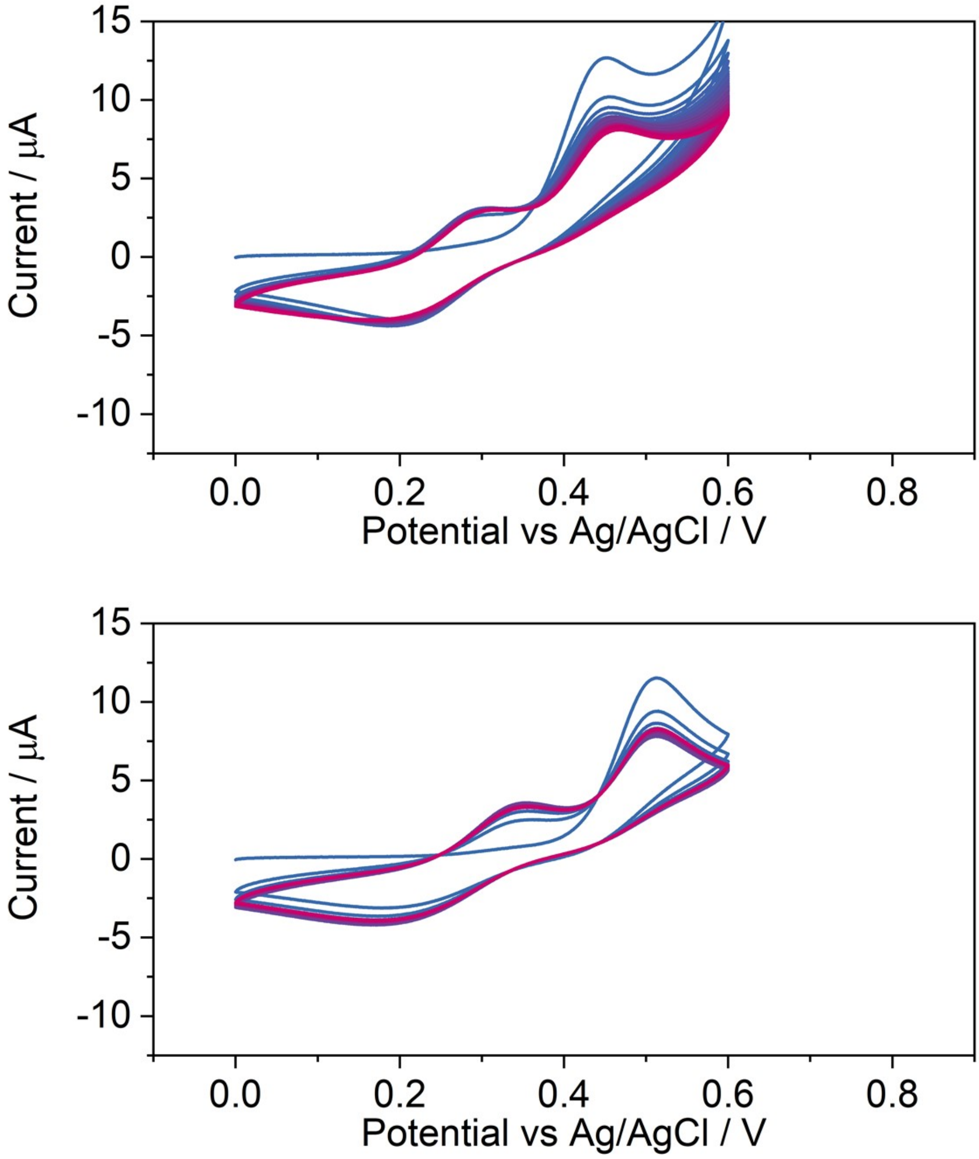

Figure S 10. *Cyclic voltammetry of* ***1 mM Ni cyclam*** $\boldsymbol{Cl_2}$ *with* ***1 M*** $\boldsymbol{K_2CO_3}$ *under* $\boldsymbol{N_2}$ *(top) and* $\boldsymbol{CO_2}$ *(bottom).*

__Table S5.__ Summary of Ni(II)/Ni(III) cyclam electrochemical characteristics between electrolytes as shown in Figure S7-8 and Figure 4.

	**Redox potential vs Ag/AgCl (mV)**	**Peak separation (mV)**	**pH of solution N_2/CO_2**
**1 M $KHCO_3$**	350 (N_2)<br>441 (CO_2)	64<br>79	8.6/7.7
**1 M KCl**	550	65	8.1/3.5
**0.1 M KPF_6**	747	74	7.6/5.0

UV-vis

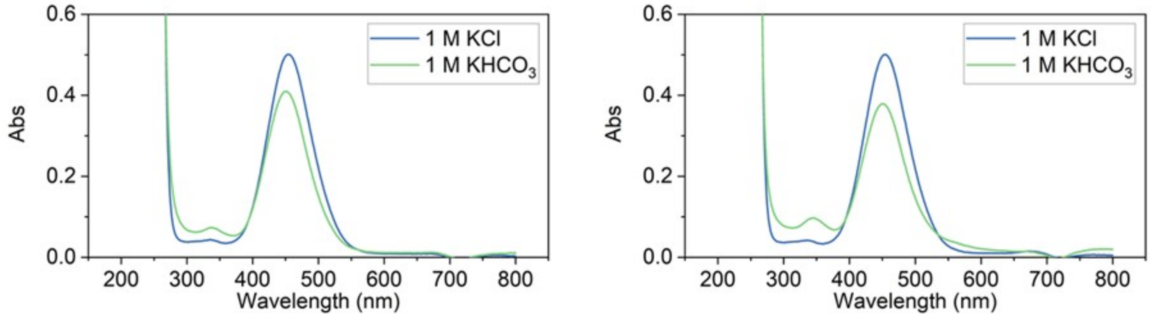

***Figure S11.** UV-vis of **10 mM Ni cyclam Cl_2** and the respective supporting electrolyte under **air** (left) and **CO_2** saturation (right).*

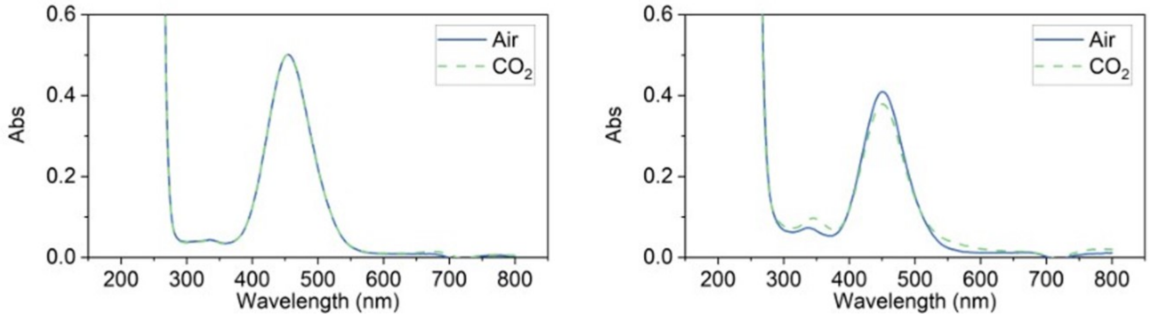

***Figure S12.** UV-vis of **10 mM Ni cyclam Cl_2** with **1 M KCl** (left) and **1 M $KHCO_3$** (right) supporting electrolytes under **air** and **CO_2** saturation respectively.*

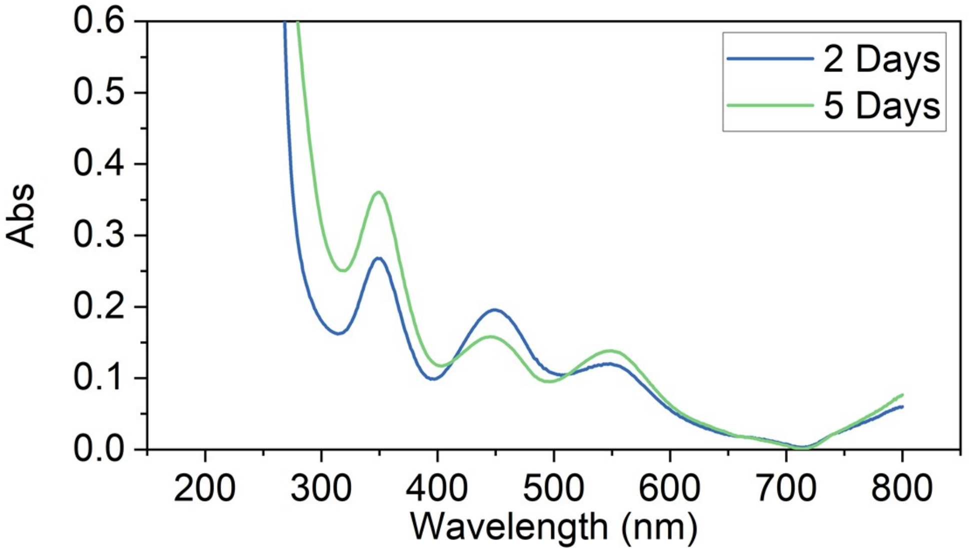

Figure S13. *UV-vis of aged **10 mM Ni cyclam*** Cl_2 *in **1 M*** $KHCO_3$ *supporting electrolyte under **air**.*

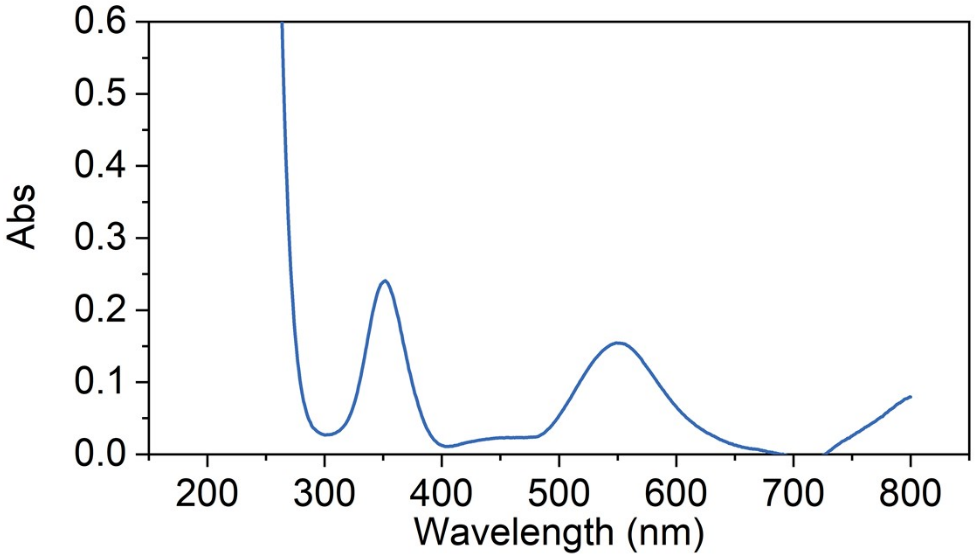

Figure S14. *UV-vis of aged **10 mM Ni cyclam*** Cl_2 *in **1 M*** K_2CO_3 *supporting electrolyte under **air**.*

Experimental

A large electrochemical flow cell with geometric electrode areas of 16 cm^2 was used to charge the electrolytes for decoupled CO_2 reduction and to test performance of the electrolytes as a cell. The cell was constructed from an outer frame of steel, polypropylene electrolyte diffusers, brass current collectors, graphite composite electrode plates, graphite felt electrodes, EPDM gaskets, and Fumatek Fumapem F-930 cation exchange membrane. The Cr PDTA negolyte was charged against $K_4Fe(CN)_6$ posolyte as a sacrificial electron source.

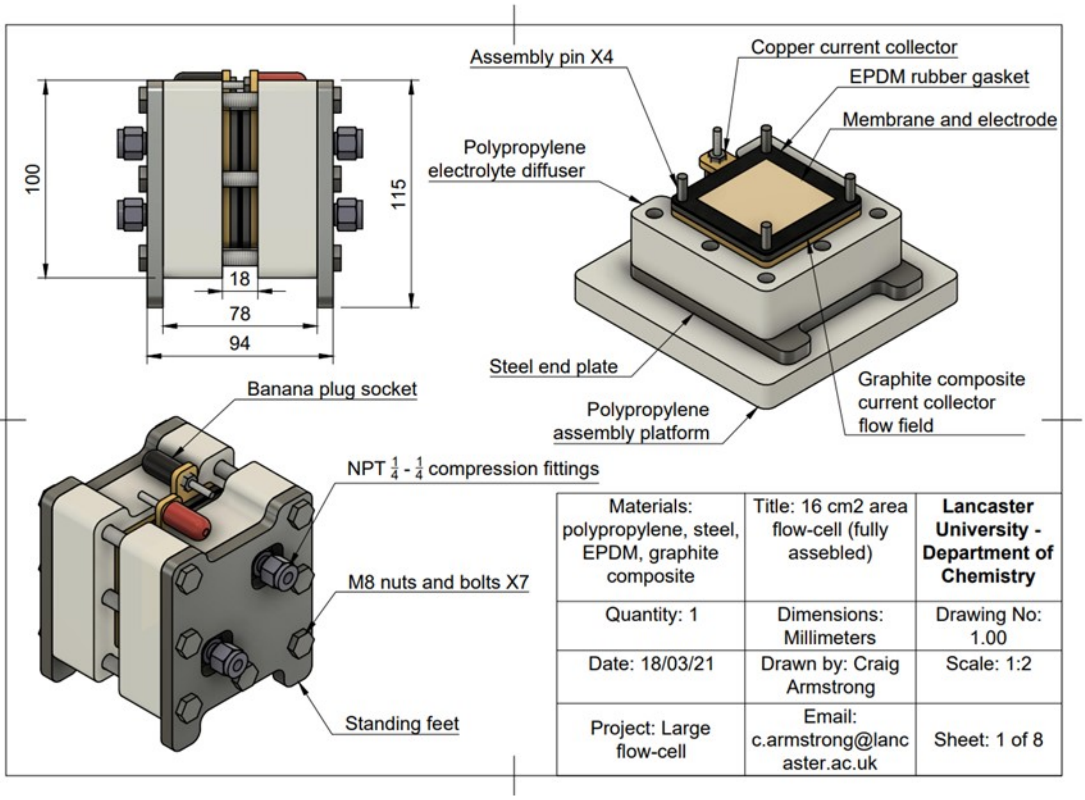

Figure S16. *Schematic representation of the 16 cm² geometric area flow cell used to access the charged state of the mediator.*

H-Cell Electrolysis

H-cell electrolysis was performed in a custom glass H-cell. The volume of the working compartment is approximately 87 mL, of which 7 mL is taken up by electrodes, 40 mL by electrolyte, and 40 mL by gas headspace. The working compartment was separated from the counter by a Fumapem F-930 cation exchange membrane. The working electrode consisted of a glassy carbon plate with a confined area of 1.2x1.2 cm, for 1.44 cm^2, onto which the catalyst was drop cast as an ink. The connecting copper wire is plastic coated to avoid contamination.

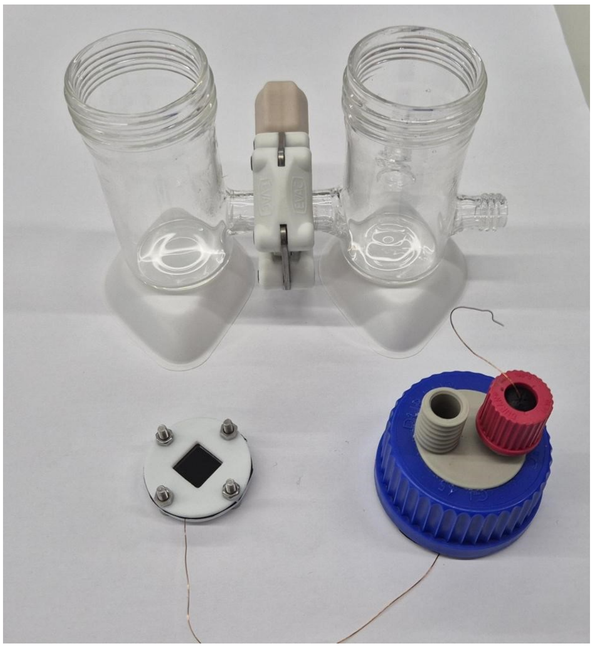

Figure S17. *Glass H-cell used for booster testing. The glassy carbon plate working electrode has a confined area of 1.2 cm x 1.2 cm. The exposed copper wire is plastic coated to avoid contamination.*

Product quantification

Calibrations were prepared to allow for the quantification of the products H_2, CO, CH_4, and C_2H_4. The gases were analysed on a Shimadzu 2030 GC system equipped with a ResTek ShinCarbon ST 80/100 column and barrier ionisation discharge (BID) detector, using helium as the eluent gas. Linear calibrations in the range 200-1,000 ppm were prepared for all four gases, with further curved calibrations in the range 2,000-100,000 prepared for H_2 and CO. Certified calibration gas standards were purchased from BOC ltd. The range of concentrations were prepared by dilution of these standards with CO_2 by the use of two gas tight syringes.

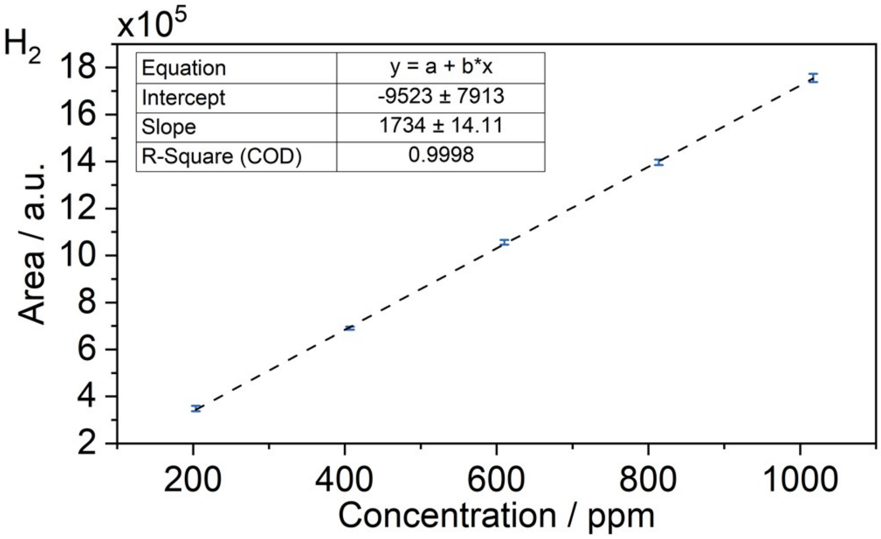

Figure S18. *Calibration plot for H_2 quantification in the range 200-1,000 ppm.*

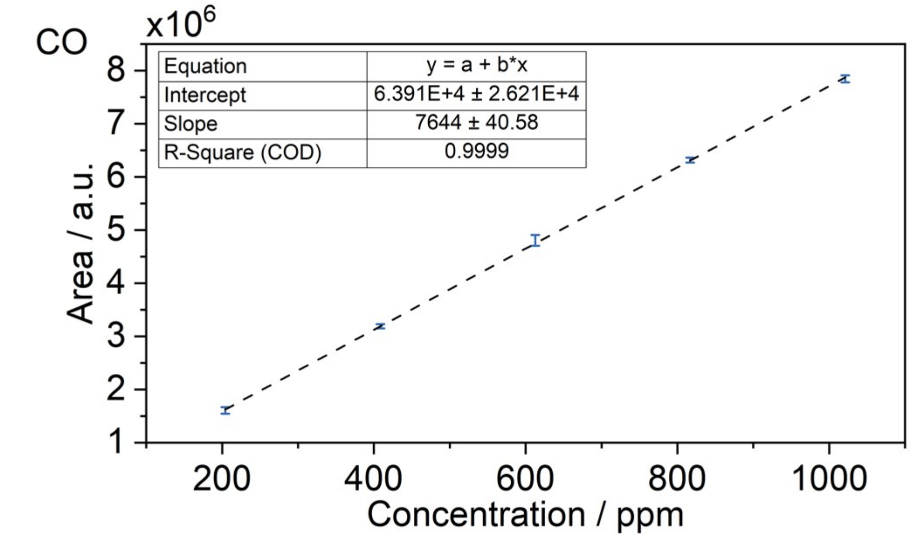

Figure S19. *Calibration plot for CO quantification in the range 200-1,000 ppm.*

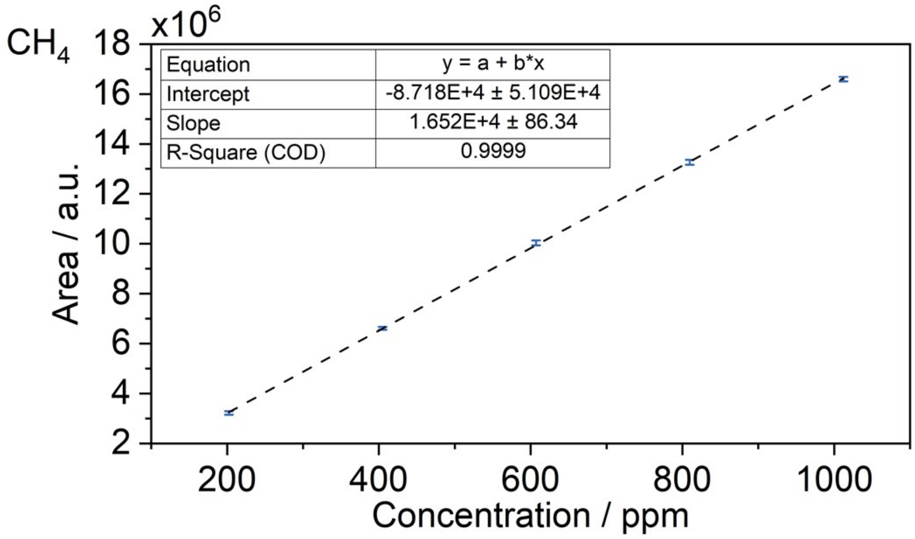

Figure S20. *Calibration plot for CH_4 quantification in the range 200-1,000 ppm.*

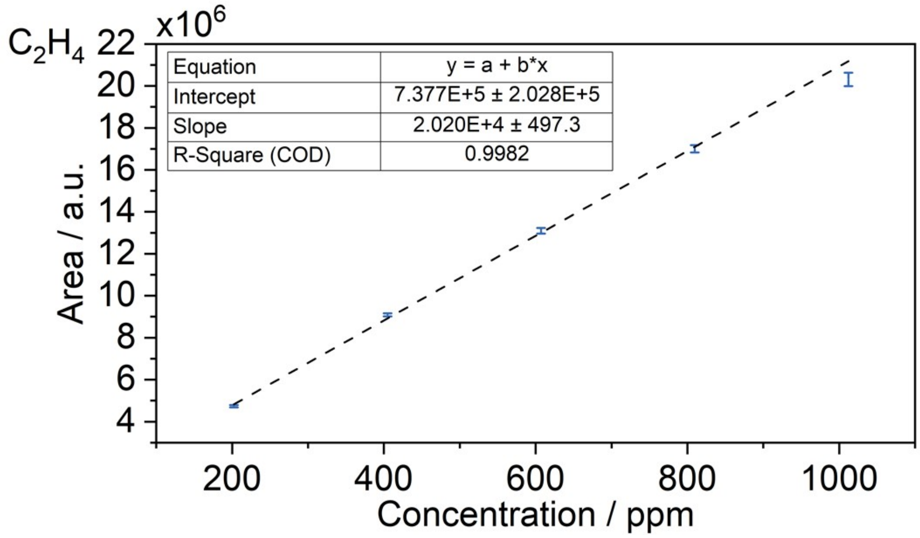

Figure S21. *Calibration plot for C_2H_4 quantification in the range 200-1,000 ppm.*

As an example, a 5-fold dilution was achieved by taking 10 mL of sample into the first syringe and 40 mL of CO_2 into the second, after which the gases were transferred between the syringes 5 times to ensure thorough mixing. The confidence of the linear calibration is high, with R^2 values >0.99 in the 200-1,000 ppm range for all gases.

The high ppm (2,000-100,000 ppm) calibrations were fitted to a curve approximated by a one phase association:

$$y = y_0 + (Plateau - y_0)(1 - e^{-kx^a})$$

Where *plateau* corresponds to the maximum y value, y_0 corresponds to the intercept, and the values of the constants *k* and *a* were optimised to minimise the error calculated from the sum of the square of the difference between real and predicted *y* values for each concentration point.

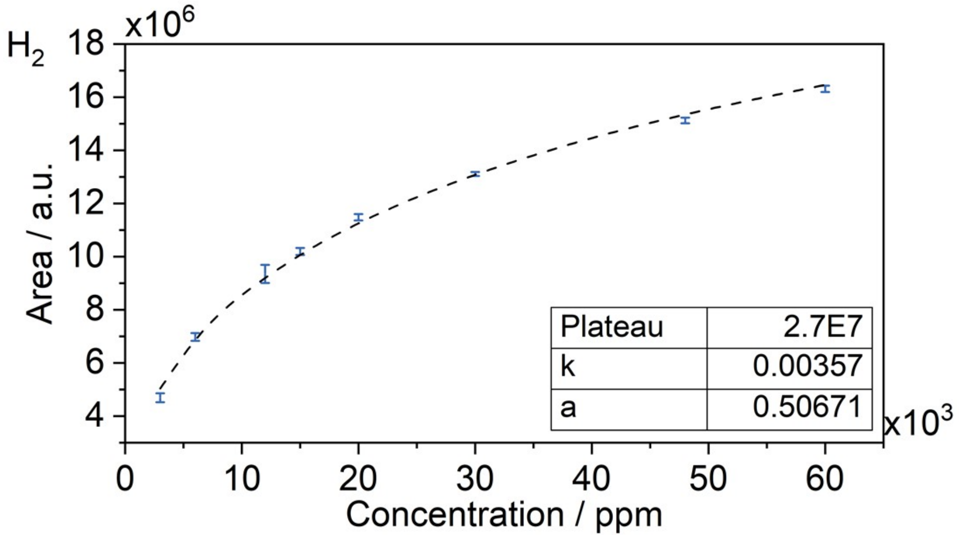

Figure S22. *Calibration plot for H_2 quantification in the range 3,000-60,000 ppm.*

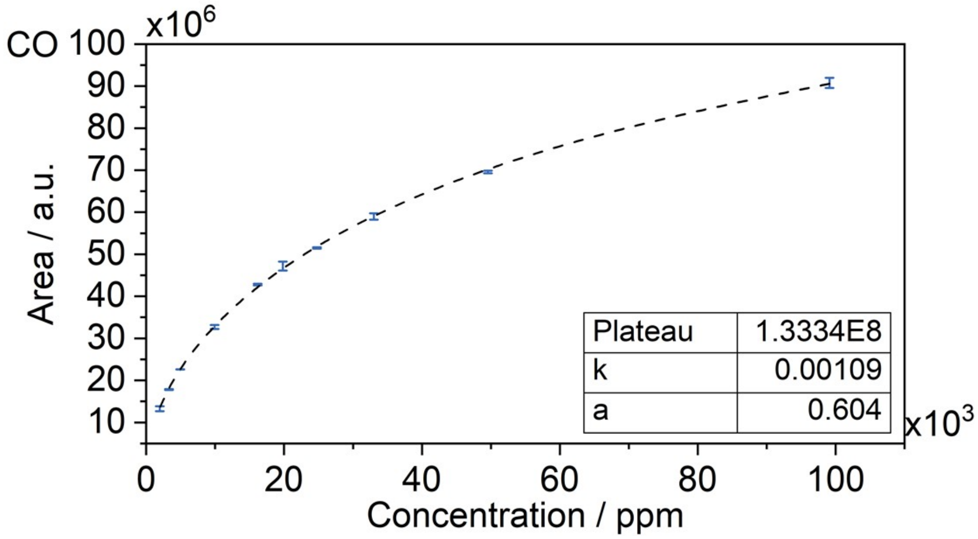

Figure S23. *Calibration plot for CO quantification in the range 2,000-100,000 ppm.*

With these calibrations, the concentration of the product gases in the headspace of the reaction vessel were measured. Knowing the total volume of each reactor and the volume of electrolyte within, the total amount of products formed can be estimated. It was assumed that the amount of each product dissolved in the electrolyte was negligible as they are all sparingly soluble.

From this, the faradaic efficiency for each product was determined.

The amount of each gaseous product in moles within the headspace was estimated;

$$Moles\ product\ [mol] \ =$$

$$\frac{\textit{Headspace concentration [ppm]}}{10^6} \times \textit{Moles of gas in headspace [mol]}$$

where the ideal gas law was used to determine the total number of moles within the headspace;

$$\textit{Total moles of gas in headspace} =$$

$$\frac{\textit{Headspace pressure } [Pa] \times \textit{Headspace volume } [m^3]}{\textit{Ideal gas constant } [J\,K^{-1}\,mol^{-1}] \times \textit{Temperature } [K]}$$

By considering the electron stoichiometry, the amount of charge needed to form each product can be calculated;

$$\textit{Charge required } [C] =$$

$$\textit{Moles product } [mol] \times \textit{Electron stoichiometry} \times \textit{Faraday constant } [C\,mol^{-1}]$$

which can then be compared to the total charge passed;

$$\textit{Charge passed } [C] =$$

$$\textit{Moles of mediator } [mol] \times \textit{Electron stoichiometry} \times \textit{Faraday constant } [C\,mol^{-1}]$$

to determine the faradaic efficiency as a quantum yield;

$$\textit{Faradaic efficiency} = \frac{\textit{Charge required } [C]}{\textit{Charge passed } [C]} \times 100$$

The same approach was employed for the liquid phase product HCOO(H), with the product moles calculated assuming a sample density of 1 kg L^{-1}

$$\text{Moles product } [\text{mol}] =$$

$$\frac{\text{Measured Concentration [ppm]}}{\text{Molecular mass } [\text{Kg mol}^{-1}]} \times \text{Total volume of electrolyte [L]}$$

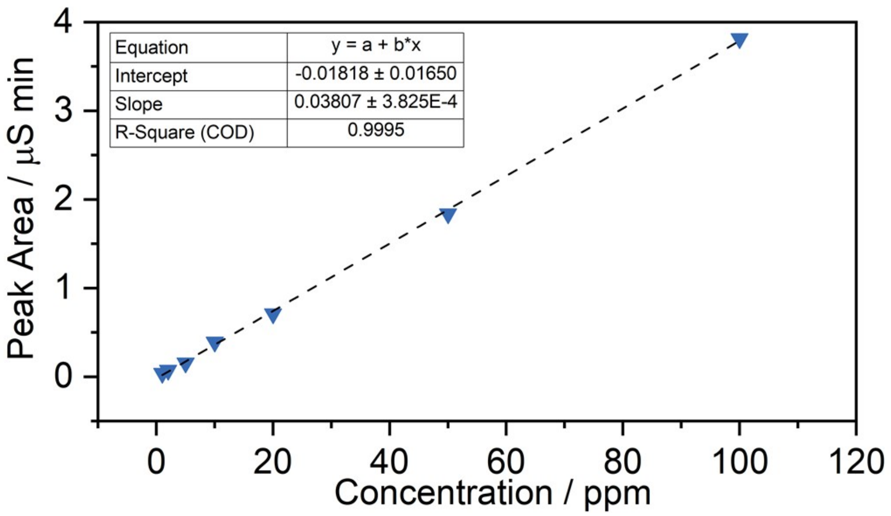

Figure S24. *Calibration plot for HCOO(H) quantification in the range 1-100 ppm.*

References

1 B. H. Robb, J. M. Farrell and M. P. Marshak, *Joule*, 2019, **3**, 2503–2512.

2 M. Potter, D. E. Smith, C. G. Armstrong and K. E. Toghill, *EES Catal.*, 2024, **2**, 379–388.

3 B. Bosnich, M. L. Tobe and G. A. Webb, *Inorg. Chem.*, 1965, **4**, 1109–1112.
